# Prevalence and severity of tinnitus in Polish otosclerosis patients qualified for stapes surgery

**DOI:** 10.1007/s00405-019-05317-8

**Published:** 2019-03-20

**Authors:** Beata Dziendziel, Piotr H. Skarżyński, Joanna J. Rajchel, Elżbieta Gos, Henryk Skarżyński

**Affiliations:** 1 Teleaudiology and Screening Department, World Hearing Center, Institute of Physiology and Pathology of Hearing, Warsaw, Kajetany Poland; 2 Heart Failure and Cardiac Rehabilitation Department, 2nd Faculty of Medicine, Medical University of Warsaw, Warsaw, Poland; 3 Institute of Sensory Organs, Warsaw, Kajetany Poland; 4 Oto-Rhino-Laryngology Surgery Clinic, World Hearing Center, Institute of Physiology and Pathology of Hearing, Warsaw, Kajetany Poland; 5 Mokra 17 Street, 05-830 Nadarzyn, Poland

**Keywords:** Otosclerosis, Tinnitus, Adults, Tinnitus Functional Index

## Abstract

**Purpose:**

To assess the prevalence and severity of tinnitus among a group of Polish patients with otosclerosis who qualified for stapes surgery. A secondary objective was to gauge the relationship between tinnitus severity and hearing thresholds.

**Methods:**

Based on the eligibility criteria, 460 adults with otosclerosis (236 women, 134 men) were included in the study. The Tinnitus Functional Index (TFI) was used to assess tinnitus severity. Hearing thresholds for air and bone conduction were established using clinical pure-tone audiometry in a soundproof cabin.

**Results:**

Based on the medical interview, tinnitus was the first symptom of otosclerosis in 35% of the participants and 65% of all patients with otosclerosis experienced clinically significant, chronic tinnitus before stapes surgery. For 59% of patients, tinnitus was a significant or severe problem. The degree of hearing loss seemed to be marginally related to the severity of tinnitus reported by the patient.

**Conclusions:**

Tinnitus is a common complaint among patients with otosclerosis, being a significant or severe problem for more than half of them. For this reason, it is worth considering in the future the implementation of standardized questionnaires for the assessment of tinnitus severity as a routine procedure in the diagnostic process of patients with otosclerosis, as well as in the postoperative period, which will be the next stage of our study.

## Introduction

Otosclerosis is one of the most complex causes of progressive hearing loss in middle-aged adults [1], although, it can also occur in older people [2] and children [3]. While its surgical and audiological aspects [4, 5] have been extensively described in the literature, knowledge of the prevalence and severity of its accompanying symptoms, such as tinnitus and balance disorders, is still limited [6]. A possible reason is that the main goal of stapes surgery has always been improved hearing.

The tendency to focus only on the audiological aspects of stapes surgery (such as closure of the air-bone gap) has been slowly changing in recent years. Increasingly, authors have begun to appreciate a wider view of the patients’ complaints, which has led to studies of the symptoms accompanying otosclerosis, which can have a considerable impact on the patient’s quality of life [7]. Apart from progressive hearing loss, tinnitus is one of the basic symptoms of otosclerosis [8]. Even in people with normal hearing, tinnitus can adversely affect day-to-day activities, being a source of emotional stress [9]. Even more commonly, subjects with a hearing loss also complain of distressing tinnitus and impaired well-being [10]. In light of these findings, it seems important to study the prevalence and severity of tinnitus in otosclerosis. Current audiometric tests are poorly related to reported tinnitus severity [11]. Another approach to assessing tinnitus complaints is to use standardized questionnaires [12]. Reliable measurements of the distress caused by tinnitus arising from different medical conditions and interventions can help improve the audiological care of patients [13, 14]. Currently, three validated tinnitus instruments are adopted into Polish: the Tinnitus Handicap Inventory published by Newman et al. [15, 16], the Tinnitus and Hearing Survey published by Henry et al. [17, 18] and the Tinnitus Functional Index created by Meikle et al. [19].

Only a few publications focusing on the assessment of tinnitus in otosclerosis have been published [20]. The majority of studies have been carried out retrospectively and have all the limitations associated with this type of study. In most cases, the main area of interest has been on whether stapes surgery eliminated tinnitus and not on its pre- or postoperative characteristics [21]. For example, based on previous research, it is impossible to estimate how many patients who undergo stapes surgery report tinnitus preoperatively, whether their tinnitus is bothersome, or what areas of life are most disturbed by tinnitus.

To help fill this considerable knowledge gap, the aim of the current study was to assess the prevalence and severity of tinnitus among a group of Polish otosclerosis patients who qualified for stapes surgery. In addition, the relationship between the air and bone conduction thresholds and tinnitus severity was evaluated.

## Materials and methods

This study included patients qualified for surgical treatment of otosclerosis between April 2017 and October 2017 in a tertiary referral center. The main eligibility criteria were:

- age ≥ 18 years;
- preoperative audiological diagnosis indicative of otosclerosis (the air-bone gap > 10 dB in pure-tone audiometry test and no stapedial reflex in the impedance audiometry test); no previous stapes surgery in the ears;
- no contraindication to take part in a questionnaire study;
- signing an informed consent for participation in the study;
- complete documentation, including medical interview, preoperative pure-tone audiometry, and tinnitus questionnaire.

The patients for whom otosclerosis was not confirmed intraoperatively were excluded.

Preoperative pure-tone audiometry was conducted in every patient according to ISO 8253-1:2010. The mean hearing thresholds for air conduction and bone conduction were determined to be 500, 1000, 2000 and 4000 Hz. The air-bone gap was defined as the difference between average bone conduction threshold and air conduction threshold. Based on the pure-tone average (PTA) of 500, 1000, 2000, and 4000 Hz, grades of hearing impairment according to the World Health Organization (WHO) guidelines were determined [22]. Based on this classification, the patients were divided into no impairment (PTA ≤ 25 dB HL), slight (PTA 26–40 dB HL), moderate (PTA 41–60 dB HL), severe (PTA 61–80 dB HL), and profound (PTA > 81 dB HL) hearing impairment groups.

Tinnitus was diagnosed as clinically significant if it occurred at least once a week and lasted at least 5 min. According to the Tinnitus Clinical Practice Guideline proposed by the American Academy of Otolaryngology-Head and Neck Surgery, tinnitus lasting more than 6 months is classified as chronic [23]. Tinnitus appearing only sporadically is classified as periodic.

Patients who were diagnosed with tinnitus were asked to fill in the Tinnitus Functional Index (TFI) [19]. The main objective of TFI is to assess treatment-related changes, comprehensively covering multiple domains of tinnitus severity. The TFI includes eight subscales: intrusiveness, sense of control, cognition, sleep, auditory, relaxation, quality of life, and emotional. The questionnaire consists of 25 items referring to the experience the patient had over the previous week. Every answer is scored from 0 to 10. The total score and scores in every subscale range from 0 to 100 points. Higher scores reflect greater severity and a more negative impact on the patient’s functioning. According to Meikle et al. [19], a TFI score below 25 points indicates relatively mild tinnitus, typically with little or no need for intervention. TFI scores from about 25–50 points suggest more significant problems with tinnitus, indicating a possible need for professional attention, and TFI scores above about 50 suggest that the tinnitus is severe enough to qualify for more aggressive efforts to provide relief.

Based on the semi-structured medical interview, data on age, gender, hearing loss duration, tinnitus duration, tinnitus localization, and sequence in which symptoms of otosclerosis occurred (hearing loss and tinnitus) were collected.

For statistical analysis, IBM SPSS Statistics v.24 software was used. A *t* test was used to compare data between the two groups. Pearson correlations were used to evaluate the relationship between the variables. Criteria provided by Fackrell et al. [24] were used to evaluate the strength of correlation: coefficients higher than 0.8 were classified as “extremely strong”, those between 0.6 and 0.79 as “strong”, between 0.3 and 0.59 as “moderate” and below 0.3 as “weak”. The statistically significant level was established at *p* < 0.05.

## Results

Among the 548 adult patients qualified for stapes surgery, 460 fulfilled the inclusion criteria. Our study group consisted of 326 (71%) women and 134 (29%) men. The age of the patients at the time of surgery ranged from 18 to 82 years (*M* = 48.0, SD = 11.5).

### Prevalence and severity of tinnitus

Based on the preoperative interview, it was found that tinnitus occurred before the hearing loss in 35% of the group; 34% of them indicated that hearing loss was the first symptom of otosclerosis. The remaining patients reported that tinnitus and hearing loss occurred at about the same time.

Among all participants, 64.8% of them—210 (71%) women and 88 (29%) men—reported preoperative clinically significant and chronic tinnitus. The average duration of tinnitus was 84 months (SD = 73.5; min 6; max 360). Bilateral tinnitus was reported by 163 patients and unilateral by 135 patients (only cases with tinnitus in the ear qualified for surgery were considered).The average score of the TFI questionnaire was 32.8 (SD = 21.3) points. According to the criteria proposed by Meikle et al. [19], 41.3% of patients had relatively mild tinnitus. However, 35.9% of patients had scores suggestive of more significant tinnitus problems, and another 22.8% had severe problems.

**Fig. 1:**
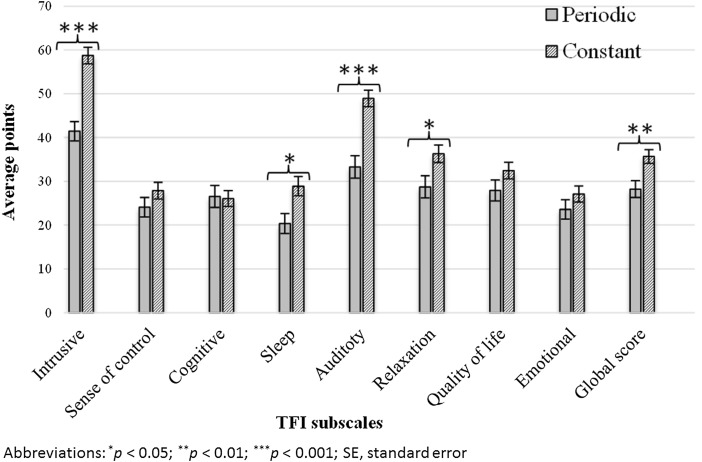
Average results of tinnitus severity for all TFI subscales in patients with periodic and constant tinnitus

Constant tinnitus in the ear qualified for surgery was reported by 183 (61%) patients and periodic by 115 (39%) patients. The two groups differed significantly in terms of severity of tinnitus in the following of TFI subscales: intrusive, sleep, auditory and relaxation (Fig. 1). The TFI global score of tinnitus severity was significantly higher in the group of patients with constant tinnitus (*t* = 2.98; *p* = 0.003).

### Degree of hearing loss

An analysis of preoperative audiometric results was performed only in the group of patients with tinnitus (*n* = 298).The average duration of hearing loss, taken to be the time between the detection of hearing loss by the patient until the time of surgery, was 9.9 years (SD = 7.9). Bilateral hearing loss was found in 197 (66%) patients. The audiometric results for the ear qualified for surgery showed mixed hearing loss in 232 people (78%) and conductive hearing loss in 66 (22%).The preoperative average hearing thresholds in the affected ear was 56.5 dB HL (SD = 16.9) for air conduction and 26.7 dB HL (SD = 13.5) for bone conduction. The average air-bone gap was 29.8 dB (SD = 9.4). An analysis of hearing loss severity based on calculated PTA showed slight impairment in 51 (17%) patients, moderate in 142 (48%), severe in 72 (24%), and profound in 33 (11%).

### Tinnitus and hearing loss

The statistical analysis revealed a weak correlation between the global score of the TFI questionnaire and average air conduction thresholds (*r* = 0.15; *p* < 0.05), and similarly for bone conduction threshold (*r* = 0.13; *p* < 0.05).With an increase in air and bone conduction thresholds, larger tinnitus severity was observed. The correlations between the TFI subscales and average air and bone conduction thresholds are shown in Table 1.

**Table 1:** Correlations between the TFI subscales and average air and bone conduction thresholds

TFI subscales	AC		BC	
	*r*	*p* value	*r*	*p* value
Intrusive	0.05	0.358	0.03	0.630
Sense of control	0.16	0.007*	0.14	0.017*
Cognitive	0.12	0.036*	0.10	0.092
Sleep	0.13	0.028*	0.12	0.024*
Auditory	0.13	0.024*	0.13	0.024*
Relaxation	0.07	0.256	0.04	0.482
Quality of life	0.16	0.007*	0.12	0.038*
Emotional	0.15	0.010*	0.12	0.033*
TFI global score	0.15	0.010*	0.13	0.029*

*AC* air conduction, *BC* bone conduction

*p < 0.05

The tinnitus severity in each hearing loss group, based on the WHO criteria, is presented in Table 2. In the group of patents with slight hearing loss, up to 45% of them had significant or severe problems with tinnitus, whereas in the group of patients with moderate to profound hearing loss, 57–64% of patients reported significant or severe problems with tinnitus.

**Table 2:** Tinnitus severity in each hearing loss group based on WHO criteria

Grades of hearing impairment	TFI global score *M* (SD)	Classification of tinnitus severity (Meikle et al. [19])		
		Mild problem with tinnitus (% of patients)	Significant problem with tinnitus (% of patients)	Severe problem with tinnitus (% of patients)
Slight (*n* = 51)	25.6 (17.8)	55	33	12
Moderate (*n* = 142)	33.7 (21.5)	39	40	21
Severe (*n* = 72)	34.4 (20.1)	36	38	26
Profound/deafness (*n* = 33)	36.7 (26.0)	43	18	39

## Discussion

Ayache et al. reported that only 6% of patients reported tinnitus as the first symptom of otosclerosis [25]. This number increased up to 74% by the time of surgery. These figures are different from our observations, where 35% of participants indicated that tinnitus occurred before hearing loss. From a clinical point of view, the occurrence of tinnitus before hearing loss (sometimes even by several years) is important. As the literature indicates, only about one in five of those who experience tinnitus seek professional services [12], even though tinnitus could be a sign of a progressive disease such as otosclerosis.

In clinical practice, special attention needs to be given to patients reporting constant tinnitus. Patients who complained about constant tinnitus are not only more annoyed by it, but also have more problems with relaxation and sleep. These problems can significantly handicap the patient in their daily activities and may cause a decrease in their quality of life [26].

According to the literature, the prevalence of tinnitus in otosclerosis is estimated to be 65–90% [27–31]. The results of our study showed that 65% of Polish patients with otosclerosis who qualified for stapes surgery experience clinically significant, chronic tinnitus. The highest figure for tinnitus prevalence, about 90%, was reported in the studies by Sobrinho et al. [27] and Rajati et al. [30]. However, the authors’ conclusions were based on a small quantity of research material. In larger studies (more than 100 participants), similar tinnitus prevalence as in our study was observed. Gristwood and Venables showed in their retrospective analysis that the presence of chronic tinnitus (lasting more than 3 months) was reported by 65% of patients [28]. Bagger-Sjoback et al. found tinnitus in 68% of their patients [29].

Because tinnitus is subjective, there are no objective diagnostic tests. Audiological evaluation of tinnitus and evidence-based interventions require the evaluation of both tinnitus perception and individual reactions to it, which can obtained using a validated questionnaire [12]. A recent review on the impact of stapes surgery on tinnitus severity revealed that only a few scientific papers have been published that include self-report tools in the evaluation of tinnitus severity [20]. For example, Dewyer et al. [32] and Chang and Cheung [33] used the TFI questionnaire to measure tinnitus severity. However, the results were presented only for the postoperative period. In the study by Bast et al., tinnitus burden was measured using the Tinnitus Questionnaire [5]. Before surgery, 23 out of 28 patients indicated they had a small or moderate problem with tinnitus, and only five patients reported severe or very severe tinnitus. Ayache et al. observed significant tinnitus in 16 (25%) patients qualified for stapes surgery [25].

In our study, the results of the TFI showed that for almost 60% of patients qualified for stapes surgery, tinnitus was a significant to severe problem. Such a high prevalence of tinnitus problems in this group can be another challenge in treating otosclerosis, as patients expect not only a hearing improvement after surgery but also a reduction in the severity of their tinnitus.

Statistical analysis between tinnitus severity and mean air and bone conduction thresholds showed only weak correlations in our study. The size of hearing loss seems to be only marginally related to the severity of tinnitus perceived by the patient. Moreover, Gristwood and Venale observed that the probability of finding an otosclerosis patient with tinnitus falls as the bone conduction and air conduction mean levels rise [28].

In Poland, the results of audiometric tests are now the gold standard for qualifying a patient for surgery in the case of suspected otosclerosis. The presence of tinnitus is not currently indicated as a sign for the surgical treatment of otosclerosis. Tinnitus is a significant or severe problem in about every second patient (especially those with mild hearing losses). In the lights of these findings, the next step will be to analyze the influence of stapes surgery on the change of tinnitus severity. A particularly interesting issue will be what group of patients can expect the biggest improvement in tinnitus after stapes surgery, and whether this reduction in tinnitus severity will be related to closure of the air-bone gap.

## Conclusion

Tinnitus is a common complaint among patients with otosclerosis, being a significant or severe problem for more than half of them. For this reason, it is worth considering in the future the implementation of standardized questionnaires for the assessment of tinnitus severity as a routine procedure in the diagnostic process of patients with otosclerosis, as well as in the postoperative period, which will be the next stage of our study.
